# A novel 20-gene prognostic score in pancreatic adenocarcinoma

**DOI:** 10.1371/journal.pone.0231835

**Published:** 2020-04-20

**Authors:** Seçil Demirkol Canlı, Ege Dedeoğlu, Muhammad Waqas Akbar, Barış Küçükkaraduman, Murat İşbilen, Özge Şükrüoğlu Erdoğan, Seda Kılıç Erciyas, Hülya Yazıcı, Burçak Vural, Ali Osmay Güre

**Affiliations:** 1 Molecular Pathology Application and Research Center, Hacettepe University, Ankara, Turkey; 2 Department of Molecular Biology and Genetics, Bilkent University, Ankara, Turkey; 3 Cancer Genetics Division, Department of Basic Oncology, Institute of Oncology, Istanbul University, Istanbul, Turkey; 4 Department of Genetics, Aziz Sancar Institute of Experimental Medicine, Istanbul University, Istanbul, Turkey; Cancer Institute, ITALY

## Abstract

Pancreatic ductal adenocarcinoma (PDAC) is among the most lethal cancers. Known risk factors for this disease are currently insufficient in predicting mortality. In order to better prognosticate patients with PDAC, we identified 20 genes by utilizing publically available high-throughput transcriptomic data from GEO, TCGA and ICGC which are associated with overall survival and event-free survival. A score generated based on the expression matrix of these genes was validated in two independent cohorts. We find that this “Pancreatic cancer prognostic score 20 –PPS20” is independent of the confounding factors in multivariate analyses, is dramatically elevated in metastatic tissue compared to primary tumor, and is higher in primary tumors compared to normal pancreatic tissue. Transcriptomic analyses show that tumors with low PPS20 have overall more immune cell infiltration and a higher CD8 T cell/Treg ratio when compared to those with high PPS20. Analyses of proteomic data from TCGA PAAD indicated higher levels of Cyclin B1, RAD51, EGFR and a lower E-cadherin/Fibronectin ratio in tumors with high PPS20. The PPS20 score defines not only prognostic and biological sub-groups but can predict response to targeted therapy as well. Overall, PPS20 is a stronger and more robust transcriptomic signature when compared to similar, previously published gene lists.

## Introduction

PDAC is among the most lethal cancer types world-wide with records of 5 year survival of less than 5% [1]. A projection of cancer incidence and death rates in US showed that pancreas cancer will become the second cause of cancer-related death by 2030 [2].

Currently the only curative option for PDAC is surgical resection (pancreaticoduodenectomy), however less than 20% of patients have resectable tumor due to the aggresiveness of the disease [3]. Standard therapy is gemcitabine and gemcitabine combinations with other drugs [4]. In recent years FOLFIRINOX and targeted EGFR inhibition by erlotinib combined with gemcitabine showed only modest improvements in response rates and overall survival [5]. A deeper understanding of the biological factors contributing to the treatment response may have the potential to improve these outcomes.

A comprehensive study of death rate trends in pancreatic cancer since 1970 revealed complexity of the disease, is largely unexplainable by known risk factors [6]. Currently the only FDA approved biomarker is CA19-9 for the management of pancreatic ductal adenocarcinoma, despite its limitations [7]. AJCC TNM staging and performance status are critical indicators of prognosis in clinical practice [8, 9]. Although multiple gene expression based prognostic prediction methods have been developed [10–16], no such test is currently used in routine practice and very few of these tests can be used as predictors of response to therapy [17], or *in vitro* sensitivity to chemotherapeutic agents in cell lines [18]. Therefore there is still much to contribute to the discovery of new risk predictors and new methodologies for prognostic assessments that can guide clinical approaches.

In this study we aimed to define a novel panel of prognostic genes for PDAC based on *in silico* analysis of microarray and RNA sequencing datasets. We thus generated a 20-gene based risk scoring method, PPS20, which can stratify patients when either overall survival or event-free survival are used as end-point measures. We show that tumors from these two patient groups are biologically distinct: tumors with high PPS20 show a higher proliferation, whereas tumors with low PPS20 have a higher rate of lymphocyte infiltration. By analyzing public cytotoxicity databases, we find that these two tumor types show differential sensitivity to specific agents.

## Methods

### Processing public datasets

RNA-sequencing raw counts and clinical data of patients with pancreatic tumors from International Cancer Genome Consortium Pancreatic Cancer Canadian and Australian (ICGC, PACA-CA and PACA-AU) cohorts were downloaded from ICGC data portal (https://dcc.icgc.org/). Primary tissue was selected as Pancreas and the Specimen Type was selected as “Primary Tumor”. Samples with sequencing based expression data “EXP-S” were downloaded. 156 and 81 patients in PACA-CA and PACA-AU cohorts, respectively, with available survival data were included in the study and raw read counts were downloaded. CPM values for each gene were calculated by the formula: (read count of the gene/total read count of sample)*1000000. TCGA PAAD RNAseqv2 Level3 RSEM normalized data and clinical data were downloaded from Broad Institute GDAC data portal (http://gdac.broadinstitute.org/). Samples with available overall survival data (n = 178) and event-free survival data (n = 133) were used. Genes which had 0 counts in more than 85% of samples were eliminated from the prognostic analyses in all RNAseq datasets. Mutational data of 150 pancreatic cancer patients within TCGA cohort was obtained from https://www.cbioportal.org/ [19]. Sample ‘TCGA-IB-7651-01” was excluded from chi square analyses since over 80% of the screened genes are altered. RNA-seq based gene counts of 51 pancreatic adenocarcinoma tissues comprising the GSE79668 dataset were downloaded from GEO (https://www.ncbi.nlm.nih.gov/geo/). CPM values were calculated using the same formula used for ICGC cohorts. Log expression was calculated as log (CPM+0.001) values for ICGC CA, ICGC AU and GSE79668 and Log2 (RSEM+0.001) for TCGA PAAD. Raw CEL files of GSE28735 dataset including 45 paired pancreatic tumor and normal tissues were downloaded from GEO and RMA normalized using BRB-array tools developed by Dr. Richard Simon and the BRB-ArrayTools Development Team. Clinical data were extracted from the series matrix file for GSE28735. 42 patients with survival data were included in survival analyses. Series matrix file of GSE21501 (n = 102) and GSE71729 (n = 123) datasets and the annotation files for GPL4133 and GPL20769, respectively, were downloaded from GEO and annotated accordingly. Clinical data was derived from the series matrix file for GSE21501. Patients with survival value “0” were eliminated from survival analyses in all datasets.

### Survival analyses and prognostic gene ranking

Log expression of all the genes and the corresponding survival data of PACA-CA, PACA-AU, TCGA and GSE21501 datasets were analyzed by an in-house R script utilizing “survival” library [20] and “coxph” functions. For each gene, Cox proportional hazards regression p value and hazard ratio were obtained separately in each dataset. Genes were ranked based on (1) Cox p value- the smaller the p value the smaller the rank, (2) Hazard ratio (HR), the greater the HR the smaller the rank (HRs smaller than 1 were included as 1/HR to make them comparable to the HRs above 1), in PACA-CA, PACA-AU, TCGA. Summation of ranks generated “ranksum” which was considered as the criteria for priority sorting genes from the smallest ranksum to the biggest. Stromal score for TCGA PAAD samples were downloaded from (https://bioinformatics.mdanderson.org/estimate/disease.html) under “RNA-Seq-V2” platform type. Residual tumor information for TCGA PAAD patients were obtained from supplementary data of the TCGA PDAC study [21]. Overall survival was used as “days to last follow-up” for censored patients and “days to death” for others, and event-free survival was calculated as “days to new tumor events” for patients with an event, “last follow time” or “days to death” for censored patients in TCGA PAAD. All survival times in days were converted to months via division by 30 for consistency in Kaplan Meier curves. Original survival values (days/months) were used for all datasets in cox regression analyses.

### Calculation of the PPS20

Log expression of the 20 genes were used in the following formula for the calculation of the prognostic score. PPS20 = ARNTL2-KANK1+MAP4K4+LDHA+SLC20A1+TRIO-ZNF557+EPS8-CBX7+RAB7A-POLR3H+STX16-PITPNA+TFG-CADPS2+ERRFI1+GSK3B-NDUFB2-C2orf42-MIA3. A PPS20 score was derived by the summation of the log expression of 11 genes related to worse outcome and subtraction of expression values of 9 genes related to favorable outcome. For genes with multiple probesets, their mean expression value was used in PPS20. For GSE28735 the score was calculated without STX16 gene as the gene was absent from the array platform. The median value of PPS20 was used as the cut-off for categorical comparisons generating “high PPS20” and “low PPS20” groups.

### Gene set enrichment analysis (GSEA)

All GSEA analyses were run using Broad Institute’s GSEA software (http://www.broad.mit.edu/gsea/) [22]. For TCGA, PACA-CA, PACA-AU and GSE71729, a pre-ranked GSEA, based on the log fold change of the expression of each gene between high PPS20 and low PPS20 groups was performed. C5.all.v6.2.symbols.gmt [Gene Ontology] was used as “Gene sets database” for all analyses. Gene sets with less than 50 genes were not included in the analyses.

### Drug sensitivity prediction analysis

To find drugs which can target low PPS20 and high PPS20 groups differentially, we used RNA sequencing data-RPKM values- produced by Cancer Cell Line Encyclopedia [23] and drug cytotoxicity data published by Cancer Therapeutic Response Portal (CTRP) [24]. CTRP dataset contains cytotoxicity data corresponding to 543 drugs screened against 38 pancreatic cancer cell lines, among other cancer cell lines. The parameter used for reporting drug cytotoxicity in this dataset is area under curve (AUC). We restricted our analyses to the compounds which are used in treatments in 16 different concentrations. Additionally not all cell lines were screened against all drugs resulting in missing values for some combinations. So we chose drugs which were screened against at least 10 cell lines (475 drugs).

We calculated PPS20 for all 37 cell lines using CCLE (Cancer Cell Line Encyclopedia) expression data and correlated it with AUC values from CTRP data using Pearson correlation (QGP1 cell line was removed as its gene expression score resulted in highly skewed data and was truly an outlier). The drugs which showed positive correlation with the score are differentially effective on high PPS20 group and drugs with negative correlation are effective on low PPS20 group.

### Re-evaluation of previously published signatures

Three previously published prognostic gene signatures for PDAC were compared to PPS20. Chen’s signature [11] is a 15 gene signature which we applied to our validation datasets (GSE62452, GSE79668). For GSE79668, staging information, which was available in T, N, and M format, was converted to TNM stage based on AJCC Staging system as a means to better compare all the validation datasets. The platform (Affymetrix Human Gene 1.0 ST Array) that was used in GSE62452, did not contain a probe for CAPN8, a gene that is part of Chen’s Signature [11]. Therefore, when calculating risk scores for Chen’s signature CAPN8 was not considered. The coefficients supplied by the authors were used and median dichotomization was used to determine the high and low risk groups. Yan’s signature [16] includes 4 genes, and the risk groups were calculated as described in the original manuscript. The third signature (Shi et al.) [17] was adapted with an approximation method. Utilizing the TCGA PAAD (Illumina HiseqV2) based outputs of Shi et al., a coefficient was generated for each gene via dividing the cut-off value of the gene that Shi et al. determined in their own article by median expression value of that gene. This coefficient was then used in other datasets to generate each dataset’s specific cut-off values by multiplication of the coefficient by the median of the specific genes. Then each gene was considered absent or present depending on that cut-off value. Absent genes were given the value 0 and present genes 1. Based on this 0–1 matrix and the cut-offs in the TCGA outputs of the paper, Shi et al. risk score was calculated as described [17]. A threshold of 1.709 was used to determine high and low risk groups.

### Statistical analysis

Kaplan-Meier curves were generated and log-rank tests and Cox regression analyses were performed using SPSS Statistics v.19 (IBM, 2010, Chicago, IL, USA). Statistical analyses were done using GraphPad Prism 5.0 (Graphpad Prism 5 Software, San Diego, CA, USA). Student’s t-tests were performed to determine differences between two groups (unpaired and not assuming equal variance-except for GSE28735, for which paired t-test was performed). Chi-squared test with Yates' continuity correction was performed using “stats” package in R Bioconductor for mutational frequency comparisons [25]. P values below 0.05 were considered statistically significant.

## Results

### PPS20 can predict clinical outcome in pancreatic cancer

**Fig 1:**
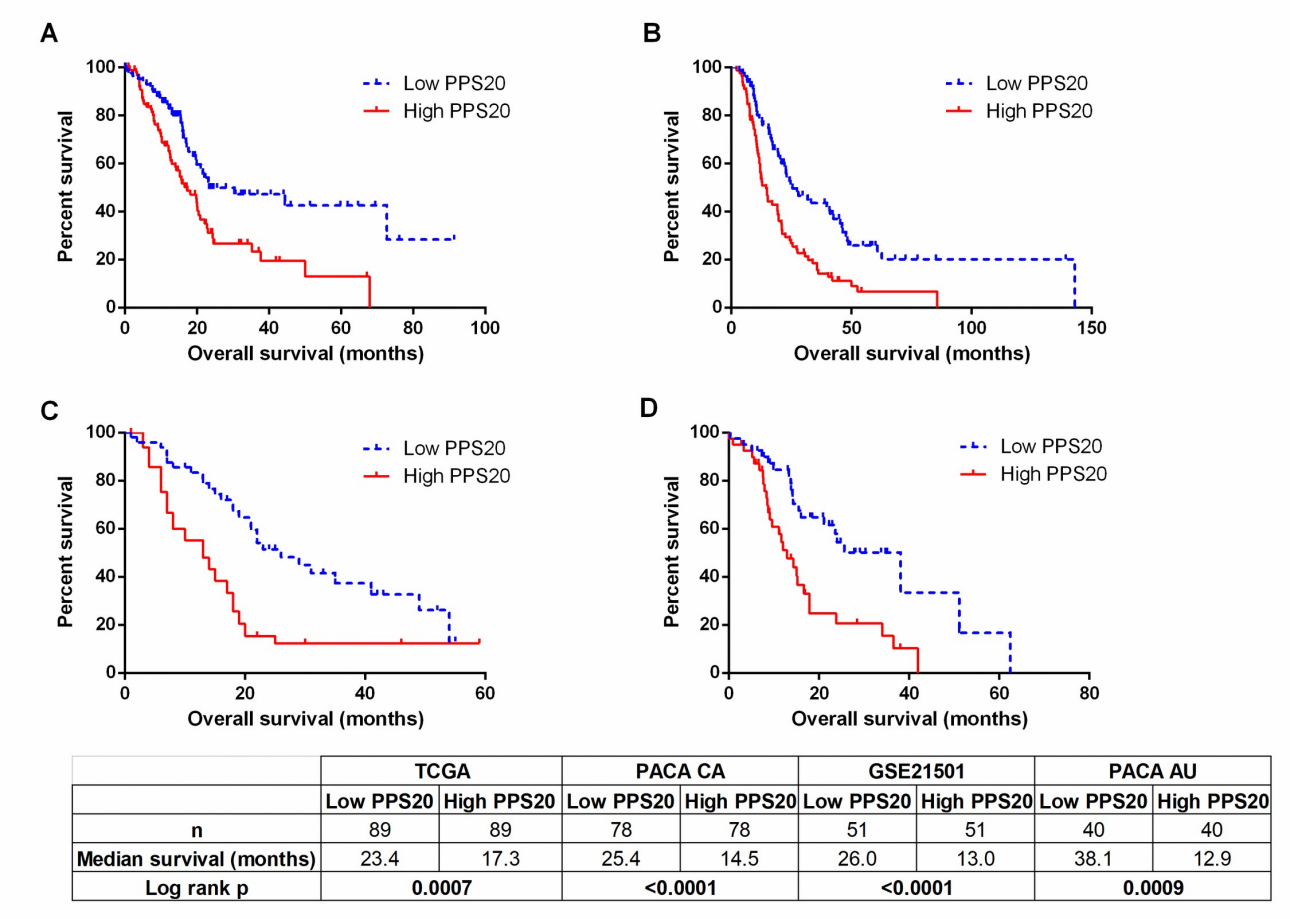
Prognostic stratification by PPS20 in discovery cohorts. Kaplan Meier graphs based on PPS20 for TCGA (A), PACA-CA (B), GSE21501 (C), PACA-AU (D). Cut-off for the score is the median in all cohorts. Statistics are given below the figure.

**Fig 2:**
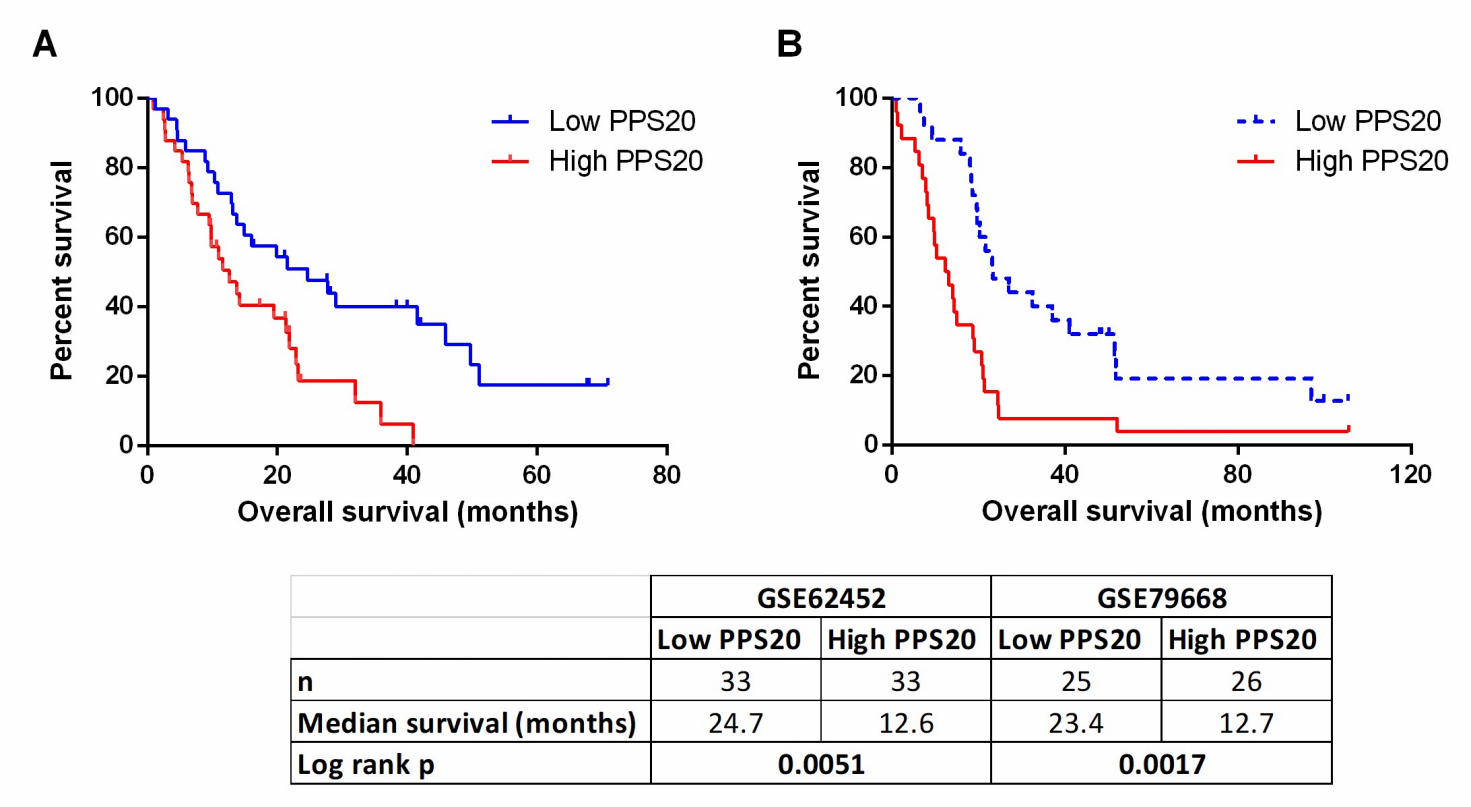
Prognostic stratification by PPS20 in validation cohorts. Kaplan Meier graphs based on PPS20 for GSE62452 (A), GSE79668 (B). Cut-off for the score is median in all cohorts. Statistics are given below the figure.

**Fig 3:**
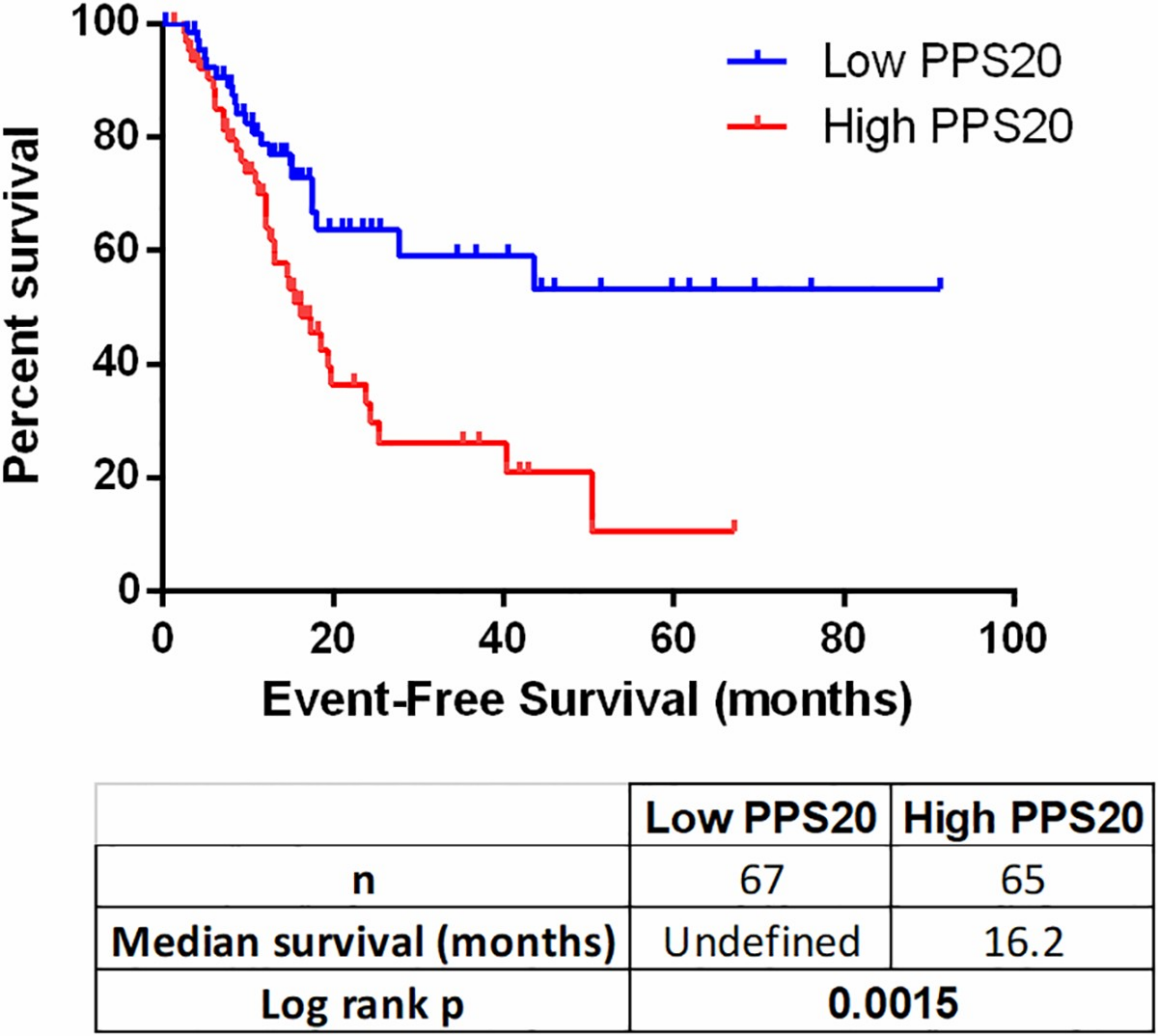
PPS20 can predict event-free survival. Kaplan Meier graph based on PPS20 in TCGA PAAD tumors. Statistics are given below the figure.

To identify individual genes related to prognosis in pancreatic adenocarcinoma we performed cox regression analyses with overall survival as an end-point measure in three discovery datasets: TCGA, PACA-CA and PACA-AU. Based on resulting HR and p values, we ranked the genes in all three datasets (see Methods). Among the top 500 genes of each dataset, 85 genes that were common in at least 2 datasets were retested utilizing the GSE21501 dataset in order to restrict the number of genes as well as to further eliminate cohort specific effects. Thus, we identified 11 and 9 genes that were significantly associated with shorter and longer overall survival, respectively (**S1 Table**). A prognostic scoring system was generated based on log expression of these 20 genes in pancreatic cancer primary tumors (see Methods for details), that we designate “Pancreatic cancer prognostic score 20 –PPS20”. As can be seen in **Fig 1**, PPS20 can stratify the patients into prognostically distinct subgroups with HR (95% CI) and p values of, 2.016 (1.362–3.088) and 0.007, 2.040 (1.498–3.089) and <0.0001, 2.416 (1.696–4.635) and <0.0001, 2.492 (1.551–5.044) and 0.0009, in TCGA, PACA-CA, GSE21501 and PACA-AU, respectively. PPS20 was then validated in GSE62452 and GSE79668 datasets, with HR (95% CI) and p values of 2.099 (1.335–4.241) and 0.0051, 2.438 (1.486–5.106) and 0.0017, respectively (**Fig 2**). A MVA that included patients without residual disease after operation and patients who did not receive targeted molecular therapy using the TCGA cohort, showed that PPS20 can predict overall survival independent of confounding factors (**S2 Table**). We then asked if PPS20 could also stratify patients when event-free survival (EFS) is used as an end-point. Indeed in the TCGA cohort, PPS20 is associated with event-free survival with an HR (95% CI) of 2.312 (1.393–3.982), p value of 0.0015 (**Fig 3**). Overall, we conclude that, PPS20 is a robust independent prognostic signature for pancreatic cancer.

### PPS20 compared to other prognostic signatures

We aimed to assess how our scoring performs when compared to previously published prognostic classifiers, Chen et. al.[11], Yan. et. al [16], and Shi et. al. [17]. A multivariate cox regression analysis which included parameters significant by univariate analyses (PPS20, Shi signature and tumor subtype) identified PPS20 as the only independent prognostic factor in GSE71729 (**Tables 1 and 2**). Similarly, PPS20 in GSE62452, PPS20 and Shi signature in GSE79668 were the only independent prognostic molecular signatures among the parameters significantly associated with survival in univariate analyses (**S3 and S4 Tables**). Therefore, PPS20 remains significant in all three analyses performed in separate cohorts; indicating it is a superior and independent prognostic classifier of PDAC.

**Table 1:** Univariate analyses.

GSE71729 (n = 123)	Nr.	HR*	P*	95% CI
**PPS20**				
High	64	**1.781**	**0.011**	**1.142–2.779**
Low	59			
**Chen Signature**				
High	64	1.173	0.471	0.760–1.809
Low	59			
**Shi Signature**				
High	45	**1.670**	**0.024**	**1.070–2.608**
Low	78			
**Yan Signature**				
High	57	0.841	0.436	0.544–1.300
Low	66			
**Tumor Subtype**				
Basal (ref)	35	**1.831**	**0.012**	**1.142–2.936**
Classical	88			
Stroma Subtype**				
Low	17	1.216	0.251	0.871–1.700
Normal	29			
Activated	77			

*Cox proportional hazards regression performed with overall survival

**Stroma Subtype’s were treated as continuous variables 1: Low, 2: Normal, 3: Activated

**Table 2:** Multivariate analyses (Backward wald).

GSE71729 (n = 123)		HR*	P*	95% CI
**Step 1**	PPS20	1.416	0.195	0.837–2.397
** **	Shi Signature	1.264	0.371	0.756–2.114
** **	Tumor Subtype	1.409	0.209	0.825–2.406
** **				
**Step 2**	PPS20	1.24	0.087	0.940–2.512
** **	Tumor Subtype	1.221	0.133	0.886–2.509
** **				
**Step 3**	PPS20	**1.335**	**0.003**	**1.142–2.779**

*Cox proportional hazards regression performed with overall survival

### Molecular characteristics of PPS20-identified PDAC sub-groups

In order to understand the biological mechanisms underlying the differences in outcome in PPS20 identified PDAC sub-groups, we performed GSEA (Gene set enrichment analysis) between the high PPS20 and low PPS20 tumors in TCGA, PACA-CA, PACA-AU and GSE71729 datasets. The gene sets with nominal p values below 0.01 and FDR q-values below 0.25 were considered enriched. Among the enriched gene sets in high PPS20 and low PPS20 groups, those common to all four datasets are listed in (**S5 and S6 Tables**), representative enrichment plots are shown in (**S1 Fig**).

**Fig 4:**
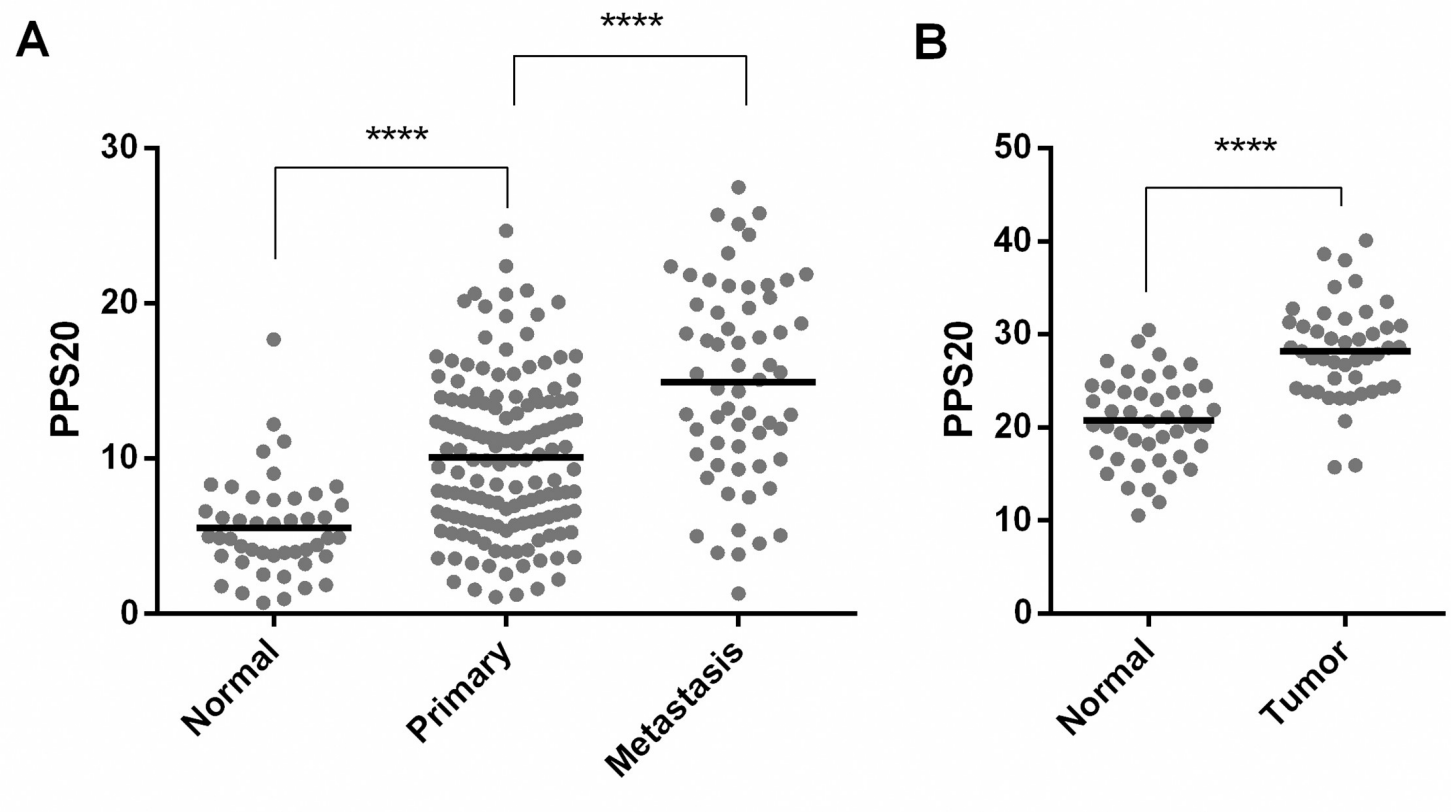
PPS20 is significantly different between normal, tumor and metastatic tissues. PPS20 in normal (n = 46), primary (n = 145) and metastasic tissues (n = 61) of GSE71729 (A). PPS20 in 45 paired tumor and normal tissues in GSE28735 (B). Horizontal lines indicate mean. Statistical comparisons were performed with unpaired and paired ttest in A and B, respectivelly. **** p<0.0001.

Enrichment of digestion and potassium channel related gene sets in low PPS20 group supports a 'normal like' phenotype, as pancreas is a ductal organ where digestive enzymes are secreted. Secretion of insulin from pancreatic beta cells is regulated by ATP-sensitive K(+) (K(ATP)) channel dependent pathways [26]. In addition HCO3- secreted by pancreatic ductal epithelial cells to duodenum neutrilizing chyme acidity is transported by multiple ion exchangers including Na+–K+–Cl– co-transporter (NKCC1) and Na+–K+-pump on the basolateral membrane [27]. Therefore the GSEA results suggest that tumors with a more favorable outcome are more differentiated, compared to those with worse outcome. In this line, we observe that the PPS20 score is lowest in normal pancreas when compared to primary tumors, and most elevated in metastatic tissues (**Fig 4**). The enrichment of protein activation cascade gene set, which mainly includes complement system proteins, might suggest a relatively higher immune activity in these tumors.

Keratinocyte differentiation, skin development and epidermis development gene sets enriched in the high PPS20 group include many genes belonging to the keratin family, among which KRT16 has been used as a basal cell marker [28, 29]; KRT17 has been shown to induce cancer stem cell-like properties in cervical cancer [30] and tumor growth, motility and invasion in gastric cancer [31], which is also associated with poor prognosis in breast cancer [32]. Formation of primary germ layer and endoderm gene sets were also enriched in tumors with high PPS20. They include many genes related to extracellular matrix, collagens, laminins, integrins, fibronectin which are known mesenchymal markers [33] and matrix metalloproteinases which are involved in tumor growth, invasion and metastasis [34], as well as HMGA2 which is known to maintain oncogenic RAS-induced epithelial-mesenchymal transition (EMT) in pancreatic cancer [35]. These results suggest that high PPS20 tumors have relatively more invasive and mesenchymal properties which is consistent with shorter event-free survival times (**Fig 3**). The same group has a lower E-cadherin/Fibronectin ratio, in line with the fact that downregulation of E-cadherin and upregulation of Fibronectin are two indicators of EMT [36] (**S2 Fig**).

In order to understand the immune involvement in the prognostic sub-groups, we analyzed TCGA PAAD tumor RNAseq data using CIBERSORT Absolute mode which enables us to assess involvement of 22 immune cell types in absolute fraction scores [37]. There is a dramatically higher “CD8 T cell–T regulatory cell” score in low PPS20 tumor together with lower scores of “Plasma cell-B cell naïve” (**S3 Fig**). These observations, together with those showing a slight increase of M2 macrophages in low PPS20 tumors are in line with an inhibition of anti-tumor immune responses. High-PPS20 tumors, however, have a larger proportion of M1 macrophages (**S3 Fig**)

The tumor-absolute score-, indicating the overall immune cell content is elevated in low PPS20 tumors (**S3 Fig**). PD-1 gene expression is significantly higher in the low PPS20 group (**S4 Fig**), when there is no significant difference in PD-L1 and CTLA-4 among PPS20 low and high groups in the TCGA dataset (**S4 Fig**).

To evaluate the mutational profile of the PPS20 groups, we utilized TCGA mutational data. Among the genes that have been altered in at least 10 patients, TP53 was the only gene which showed significantly different mutation frequency between PPS20 groups; with 79.3% mutated in high PPS20 and 56.4% mutated in low PPS20 tumors (**S7 Fig**). A multivariate analyses of PPS20 and TP53 mutation status resulted cox regression outputs of p = 0.02 HR = 1.74 (95% CI: 1.07–2.83) for PPS20 whereas no significance obtained for TP53. Overall these results indicate that there is no relationship between PPS20 and mutational status except for TP53, and PPS20 can predict prognosis independent of TP53 mutation status.

### PPS20 as a predictor of response to targeted therapy

When we stratified TCGA PDAC patient data by PPS20, we observed that high PPS20 patients who received molecular targeted therapy had significantly better prognosis compared to patients who did not receive the same therapy (**S5 Fig**) while no significant difference was observed in the low PPS20 group, or in case of radiation therapy response (**S5 Fig**). Unfortunately, the specific drug information used in molecular targeted therapy of these patients is not given in TCGA. We also noted that EGFR, RAD51, Cyclin B1 protein level expressions are significantly higher in high PPS20 patients (**S2 Fig**), indicating a proliferative activity in this group. Overall, these results show that the PPS20 score can be an identifier of response to molecular targeted therapy in PDAC, especially for high PPS20 patients.

### Identification of compounds selectively targeting individual risk groups

In order to identify compounds targeting low PPS20 and high PPS20 groups, the score was applied to CCLE pancreatic cancer cell lines. Pearson correlation analyses between the score and AUC of drugs in CTRP database resulted in the discovery of 40 drugs (S7 Table, most significant 5 drugs are shown for each group). The most effective drug for the high PPS20 group was BIRB-796, which is a p38 MAPK inhibitor (S6A Fig). Among the drugs that are effective on low PPS20 group, Ouabain was the most significant which is the inhibitor of the Na+/K+-ATPase (S6B Fig). This observation is also in line with our GSEA results showing enrichment for potassium ion transport and potassium channel activity.

## Discussion

In contrast to other cancer types like colon and breast for which multiple molecular tests are available for risk prediction and/or molecular subtyping, such as PAM50 [38], MammaPrint [39], Oncotype Dx Breast [40], Oncotype Dx Colon [41], there are limited number of molecular signatures defined for prognostication of pancreatic cancer in the literature and none are available to guide clinical therapeutic decisions in practice. A major reason for this is the lack of validation of the present signatures in multiple patient cohorts. As the number of publically available high-throughput transcriptomic data has increased over time, it became possible to include higher number of patient samples/cohorts into biomarker discovery methodologies that enabled identification of more robust biomarkers which are not cohort specific. Using this strategy, we previously identified two mRNA based biomarkers, ULBP2 and SEMA5A, for the prognostication of colon cancer [42]; and identified an independent gene panel for prediction of prognosis in both diffuse and intestinal type gastric cancer (unpublished data); which shows that the growing transcriptomic data enables discovery of such biomarkers which could have been missed when less patients studied.

Therefore, in this study, we aimed to enlarge the number of *in silico* cohorts and used 4 discovery and 2 validation PDAC gene expression datasets, including data from RNA sequencing and multiple microarray platforms. Indeed, eighteen of 20 genes identified in this study were not included in previously published prognostic gene signatures; two, ARNTL2 and SLC20A1, were used in Shi et al.[17], and Haider et al.[10], respectively. Our results show the robustness of PPS20 even when different assay platforms are used. Although tumor stage (I-IV), differentiation status (poor-moderate-well) and clinical characteristics as such as age and gender varied highly among the datasets analyzed, PPS20 could stratify prognostic subgroups independent of clinical confounding parameters in all cohorts. Indeed these results clearly will be clinically more relevant when validated *ex vivo* in a large patient cohort.

We compared three different prognostic signatures generated for PDAC to PPS20. We applied these predictive signatures as they were described in their respective publications with modifications as described in the methods section. Two of these (Chen et al (Moffit)[11] & Yan et al[16]) were predictors of overall survival. The third signature that was compared to PPS20 was Shi et al’s signature [17] which predicts recurrence free survival. Although the coefficients that were determined from the original study were utilized in determining the cutoff values for different datasets, the expression values of each gene might not be identical to those used in the original publication and this in turn might have caused some genes that would normally be assigned a value of 1 to have a value of 0. Therefore our application of the Shi signature should be considered only an approximation. In summary, although the aforementioned signatures are, in our analyses, not superior to PPS20, they would need to be further validated utilizing identical tumor samples for a conclusive analysis.

The risk groups identified in this study have distinct molecular features. In high PPS20 group, we found increased protein level expression of Cyclin B1, which is a marker of cell proliferation and as well as DNA repair [43] which is consistent with increased RAD51 which is involved in double stranded break repair, tumor progression and resistance to anti-cancer treatments [44]. TRIO, SLC20A1, MAP4K4 and ERRF1 genes which are upregulated in high PPS20 patients, were also shown to be involved in cellular proliferation and/or tumor growth [45–48]. EGFR, which is one of the major drivers of cell proliferation [49], was also higher in the high PPS20 group at the protein level. EGFR expression has been detected in 25–90% of the pancreatic adenocarcinomas in different studies and is associated with stage, metastasis, poor differentiation and survival [50].

We find a differential response to molecular targeted therapy, in high PPS20 group. When the patients are first stratified by PPS20, we have showed that high risk patients who were treated with targeted therapy have significantly longer overall survival (Log rank HR: 5.104, p value<0.0001) compared to untreated group, with median survivals of 23 and 6.1 months, respectively. Therefore PPS20 stratification can enable a striking contribution to prediction of targeted therapy success, from an overall survival benefit of 0.33 months to 16.9 months. Overall, a better prediction of responders to molecular targeted therapy can be achieved when *ex vivo* validations are performed for PPS20 and when specified agents are known.

E-cadherin/Fibronectin ratio is slightly lower in high risk group, indicating mesenchymal properties, in line with the association of high PPS20 with shorter event-free survival. Consistently, EPS8, one of the genes related to worse prognosis in PPS20 which functions as part of the EGFR pathway also regulates actin cytoskeleton and promotes EMT [51, 52]. Among the genes associated with shorter survival in PPS20, TRIO, LDHA, MAP4K4 and ARNTL2 contribute to cellular motility/invasiveness/tumor aggressiveness and thus can also be contributing to shorter event-free survival [45, 48, 53, 54]. Similarly, LDHA, EPS8, SLC20A1 and ARNTL2 expression are also related to metastasis or shorter survival in pancreatic cancer [10, 17, 55, 56].

We interpret the dramatically higher “CD8 T cell–T regulatory cell” score in low PPS20 tumors to show the presence of larger amounts of CD8 CTLs in these tumors. The higher scores of “Plasma cell-B cell naïve” in high PPS20 tumors, suggesting a stall in B cell activation, might support an ineffective CTL response in this tumor group. The dominance of M2 macrophages which are known to inhibit anti-tumor T cell responses [57] in low PPS20 tumors, further supports this hypothesis. On the other hand a larger M1 proportion in high-PPS20 tumors correlates with their mesenchymal phenotype, as pro-inflammatory cytokines produced by M1 macrophages are known to promote EMT [58]. As PD-1 gene expression is significantly higher in the low PPS20 group, despite the absence of a significant difference in PD-L1 and CTLA-4 among PPS20 low and high groups, these findings cumulatively suggest that PPS20 low tumors might benefit from anti-PDCD therapy.

GSEA results indicate an enrichment of complement system proteins which can promote T cell activation and maturation [59]. Our GSEA analysis also indicated a “normal like”, more differentiated phenotype of low PPS20 tumors with an enrichment of digestion and ion channel transport related gene sets. Among genes upregulated in this group, CBX7, MIA3 and KANK1 have been shown to have tumor suppressive activities including roles in inhibition of cellular motility/migration and/or proliferation, and induction of cell cycle arrest in various malignancies [60–67]. PITPNA which we found as a good prognostic indicator, is suggested as favorable prognostic marker in pancreatic, endometrial and renal cancers in the Human Protein Atlas (www.proteinatlas.org) [68], and its overexpression was associated with longer survival in PDAC [69]. Human protein atlas also suggests POL3HR and C2ORF42 are favorable prognostic markers in pancreatic cancer.

Via analyzing cell line drug cytotoxicity data we identified a list of compounds which are selectively effective on either low PPS20 or high PPS20 tumors. The most significant associations were with BIRB-796, a p38 MAPK inhibitor for high PPS20 group and quabain which is a Na+/K+ATPase inhibitor for low PPS20 group. The role of p38 MAPK in regulating tumor cells’ proliferation, apoptosis and metastasis has been previously extensively reviewed [70]. Specifically in pancreatic tumors, p38 MAPK expression has been shown to be increased compared to normal tissues, and targeting p38α has shown to inhibit pancreatic cancer cell proliferation; in addition a higher expression level in tumors was associated with worse overall survival [71]. Another study showed that activation of p38 results in proliferation, invasion, and metastasis of pancreatic cancer cells leading to worse prognosis and its suppression prevents the progression of pancreatic cancer [72, 73]. Additionally, under hyperglycemic conditions, increased 38 MAPK signaling is also responsible for epithelial to mesenchymal transition of pancreatic cells and upon p38 MAPK inhibition, these cells reverted back to a relative epithelial phenotype and tumor volume was decreased [74]. Increased expression of p38-MAPK has been also related to chemotherapy resistance in human gastric cancer cells [75]. The compound identified by our analysis has previously been shown to inhibit all 4 types of p38 MAPK isoforms both *in vivo* and *in vitro* [76] and has been shown to increase the efficiency of other chemotherapeutic agents in drug resistant models [77]. Here in our analysis, we found that BIRB-796 (p38α inhibitor [78]) differentially target high PPS20 group.

In esophageal squamous cell carcinoma, overexpression of Na+/K+ ATPase is associated with severity of the disease and is also reported in medulloblastoma, glioblastoma, melanoma, hepatomas, and non-small-cell lung cancer [79], and in breast cancer, it is reported to increase invasion of endocrine resistant cancer cells [80]. Although Qubain causes Na+/K+ATPase to interact with Src and EGFR, and can actıvate ERK1/2, it also results in growth arrest in human breast cancer cells, possibly by increasing the expression of p53 and p21 [81]. Its antiproliferative effects have been also shown in prostate cancer cells, and in pig kidney epithelial cells [82–84]. Since our GSEA results showed enrichment of potassium ion transport and potassium channel activity in low PPS20 group, an inhibitor of a Na+/K+ pump can be a potential drug for this group of patients subsequent to *in vitro* and *in vivo* validation.

## Conclusions

We identified a gene signature composed of 20 prognostic genes (PPS20), and a score generated based on expression of these genes. PPS20 identifies both prognostically and biologically distinct sub-groups among PDAC tumors, and has potential as a predictive marker of response to molecular targeted therapy for PDAC.

## Supporting information

S1 Fig Examples to genesets enriched in low PPS20 and high PPS20 groups in TCGA and PACA CA datasets. (TIF)

S2 Fig Proteome analysis of pancreatic tumors in TCGA. Normalized protein expression values shown for low PPS20 (n = 53) and high PPS20 (n = 70) TCGA PAAD primary tumors. Horizontal lines indicate mean. Unpaired ttest results are given. **p<0.01, *p<0.05.
(TIF)

S3 Fig CIBERSORT based immune cell content of PPS20 based groups in TCGA. Immune cell fraction scores were obtained for each tumor sample using https://cibersort.stanford.edu. The samples with a deconvolution p value below 0.05 were included in the analysis. The differences between two immune cell fractions are given for “T cells CD8 and Tregs”, “M1 and M2 macrophages”, and “Plasma cells and naive B cells”. Unpaired ttest results are given. **** p<0.0001, ***p<0.001
(TIF)

S4 Fig Patients with low PPS20 have elevated PD-1 expression. Log transformed RSEM values plotted for low PPS20 (n = 89) and high PPS20 (n = 89) TCGA PAAD primary tumors. Horizontal lines indicate mean expression. Unpaired ttest was performed. *p<0.05.
(TIF)

S5 Fig High PPS20 group shows a favorable outcome when treated with targeted molecular therapy. Kaplan Meier graphs stratified by PPS20 in TCGA PAAD comparing patients who received and did not receive radiation therapy and molecular targeted therapy. Statistics are shown below the figure.
(TIF)

S6 Fig BIRB-796 sensitivity is negatively correlated with PPS20 and can preferentially target cells with high PPS20 **(A).** Ouabain shows the opposite pattern **(B)**.
(TIF)

S7 Fig Comparison of mutation frequencies between PPS20 groups in TCGA. The genes which have been altered in at least 10 patients are shown. Chi-squared test with Yates' continuity correction was performed for each gene (synonymous variants excluded). Blue: No alterations, Yellow: Synonymous variants, Red: Nonsynonymous variants. * indicates a p value smaller than 0.05.
(TIF)

S1 Table Genes involved in PPS20. (DOCX)

S2 Table Cox regression analyses of PPS20 and clinicopathological parameters–OS. (DOCX)

S3 Table **A:** Univariate Analyses. **B:** Multivariate Analyses (Backward Wald).
(DOCX)

S4 Table **A:** Univariate Analyses. **B:** Multivariate Analyses (Backward Wald).
(DOCX)

S5 Table Gene sets enriched in high PPS20 tumors. (DOCX)

S6 Table Gene sets enriched in low PPS20 tumors. (DOCX)

S7 Table **A**: 5 drugs targeting cells with ‘high PPS20’. Negative correlation between PPS20 and drug cytotoxicity data (AUC) shows drugs targeting ‘high PPS20’ group. **B:** 5 drugs targeting cells with ‘low PPS20’. Positive correlation between PPS20 and drug cytotoxicity data (AUC) shows drugs targeting ‘low PPS20’ group.
(DOCX)

## Abbreviations

PDAC: Pancreatic ductal adenocarcinoma
PPS20: Pancreatic cancer prognostic score 20
ICGC: International Cancer Genome Consortium
PACA-CA: Pancreatic Cancer Canadian
PACA-AU: Pancreatic Cancer Australian
GEO: Gene Expression Omnibus
TCGA: The cancer genome atlas
PAAD: Pancreatic Adenocarcinoma
HR: Hazard ratio
RPKM: Reads per kilo base per million mapped reads
CTRP: Cancer Therapeutic Response Portal
AUC: Area under curve
CCLE: Cancer Cell Line Encyclopedia
GSEA: Gene set enrichment analysis
