# Supplementary figures and images for: A novel 20-gene prognostic score in pancreatic adenocarcinoma
Source: PLoS One. 2020 Apr 20;15(4):e0231835. doi: 10.1371/journal.pone.0231835 (PMC7170253; doi:10.1371/journal.pone.0231835)

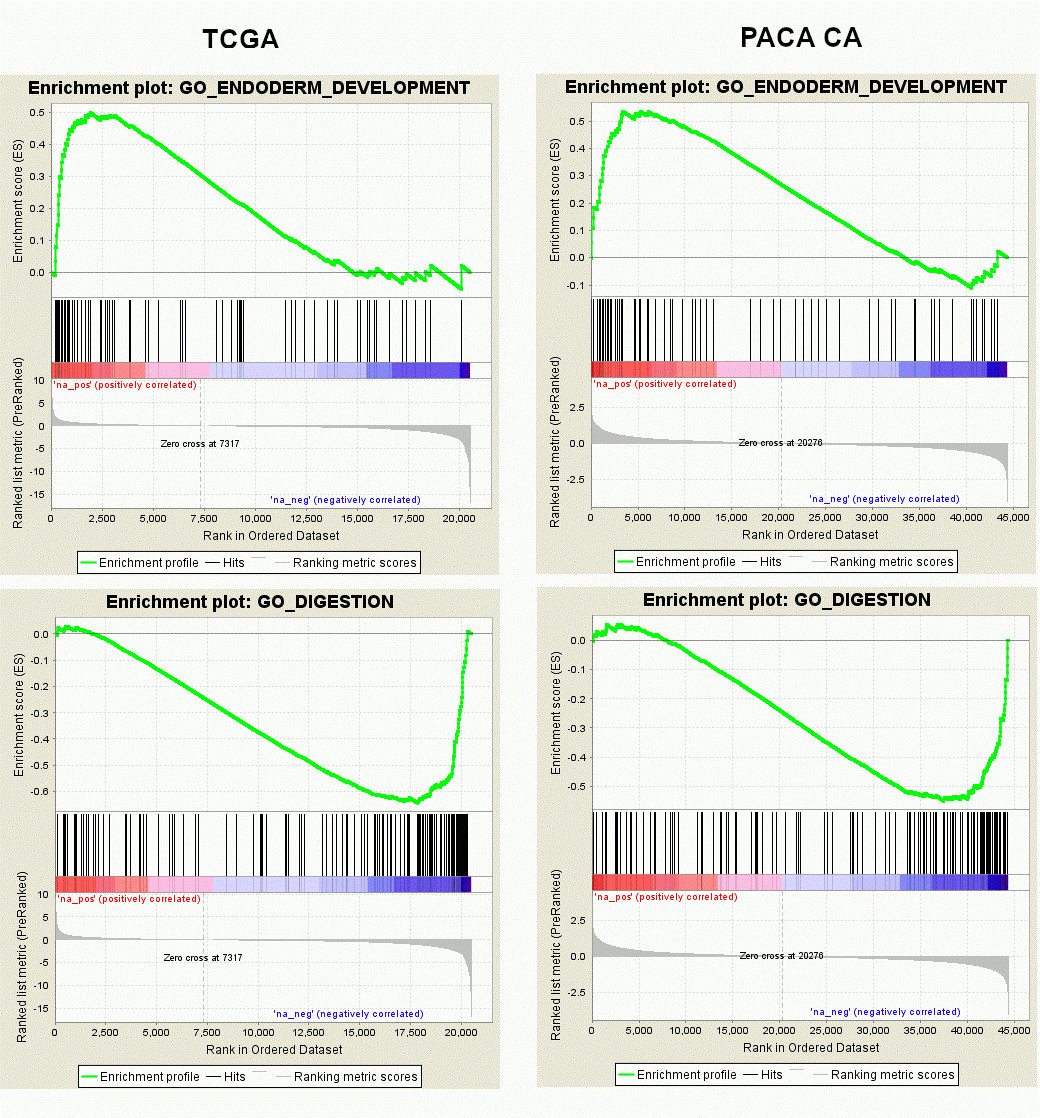

Supplement: S1 Fig — (TIF) [file pone.0231835.s001.tif]

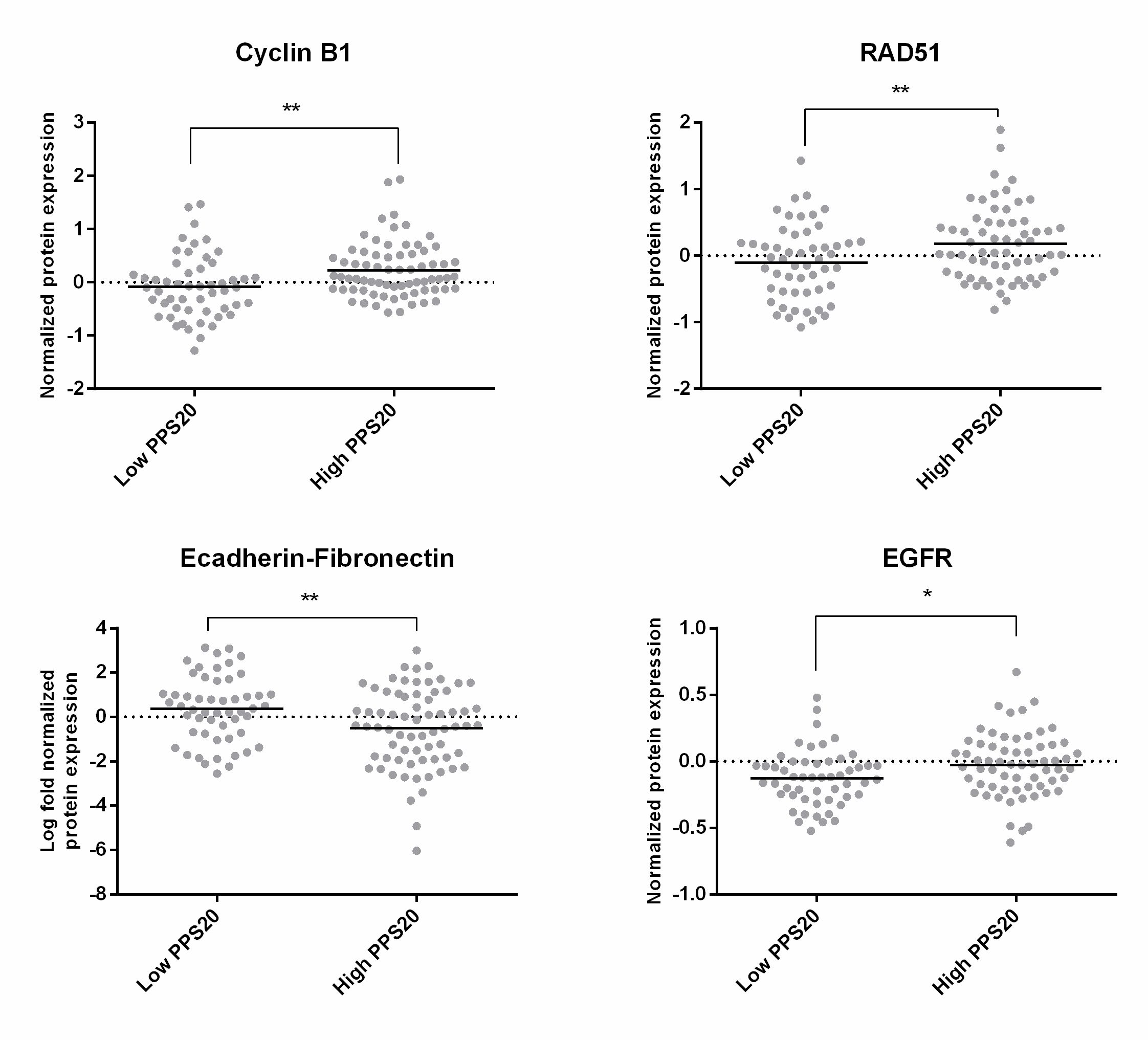

Supplement: S2 Fig — Normalized protein expression values shown for low PPS20 (n = 53) and high PPS20 (n = 70) TCGA PAAD primary tumors. Horizontal lines indicate mean. Unpaired ttest results are given. **p<0.01, *p<0.05. (TIF) [file pone.0231835.s002.tif]

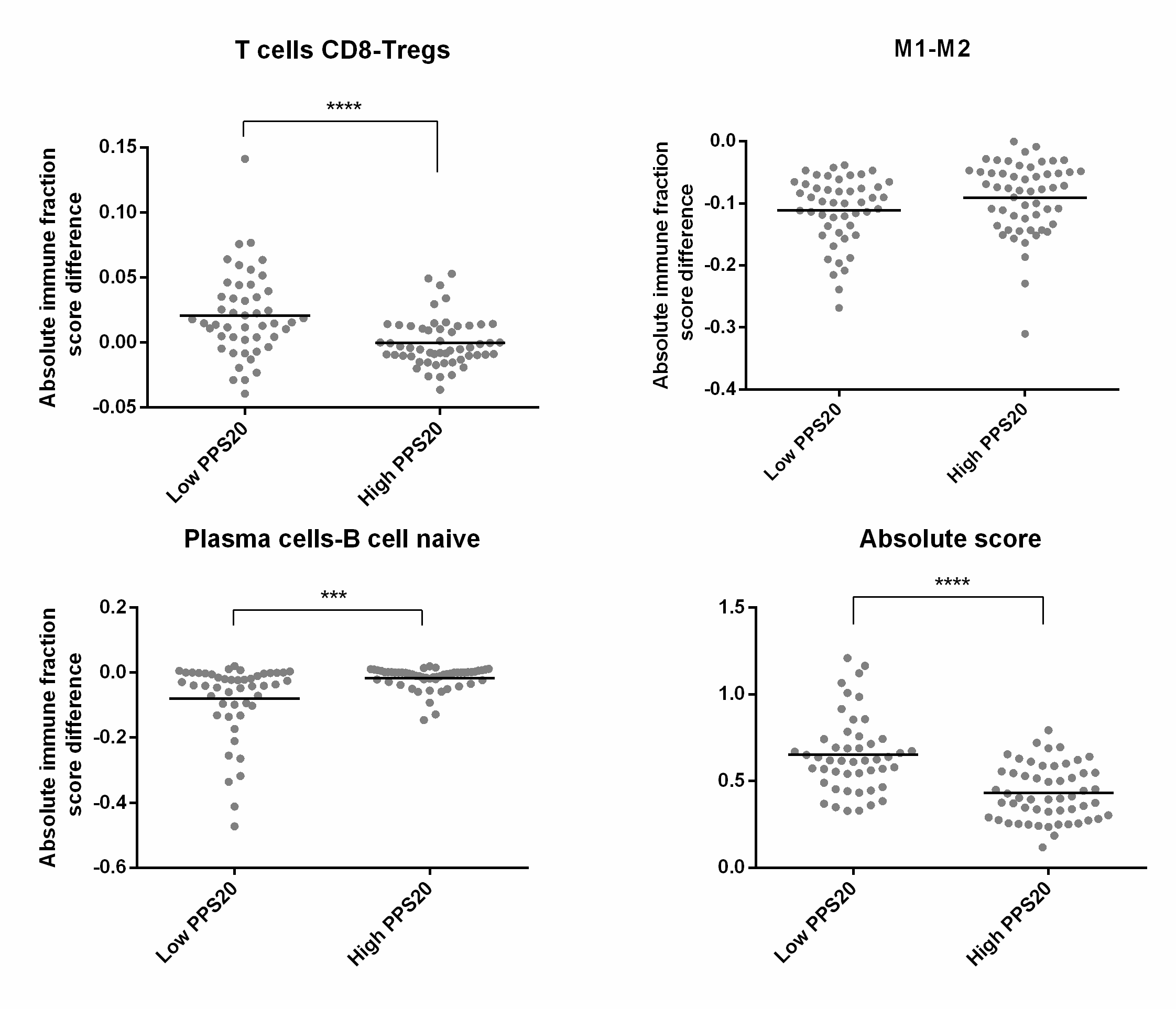

Supplement: S3 Fig — Immune cell fraction scores were obtained for each tumor sample using https://cibersort.stanford.edu. The samples with a deconvolution p value below 0.05 were included in the analysis. The differences between two immune cell fractions are given for “T cells CD8 and Tregs”, “M1 and M2 macrophages”, and “Plasma cells and naive B cells”. Unpaired ttest results are given. **** p<0.0001, ***p<0.001 (TIF) [file pone.0231835.s003.tif]

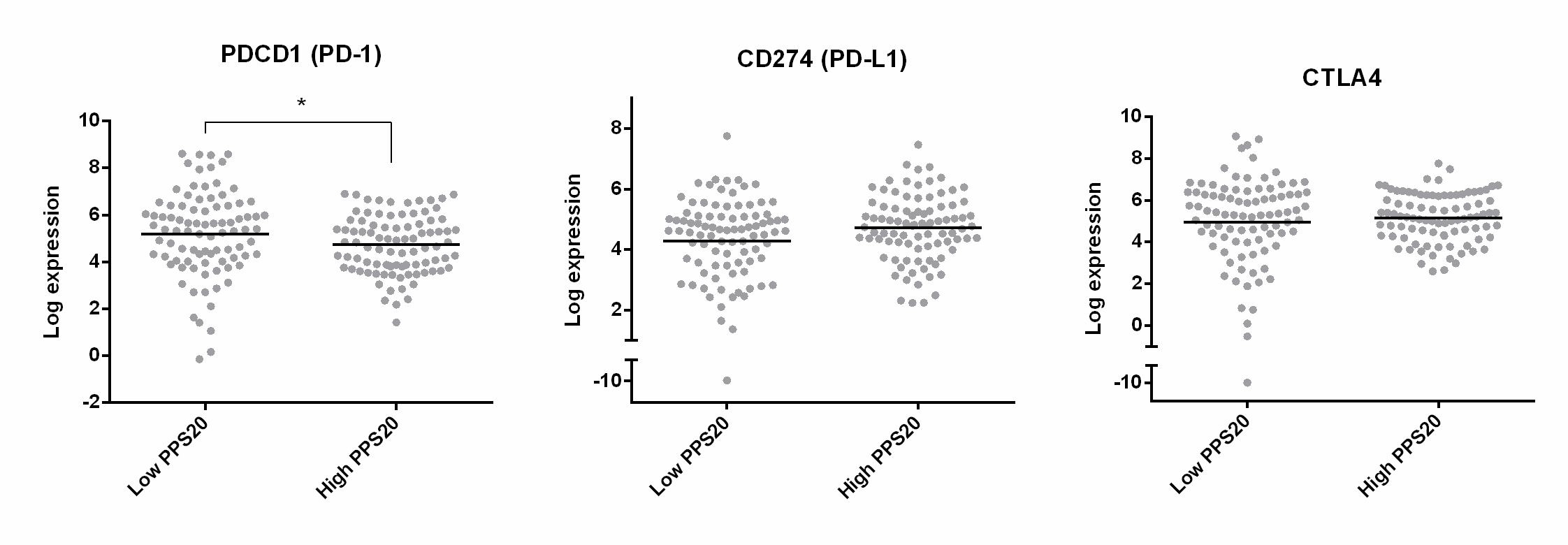

Supplement: S4 Fig — Log transformed RSEM values plotted for low PPS20 (n = 89) and high PPS20 (n = 89) TCGA PAAD primary tumors. Horizontal lines indicate mean expression. Unpaired ttest was performed. *p<0.05. (TIF) [file pone.0231835.s004.tif]

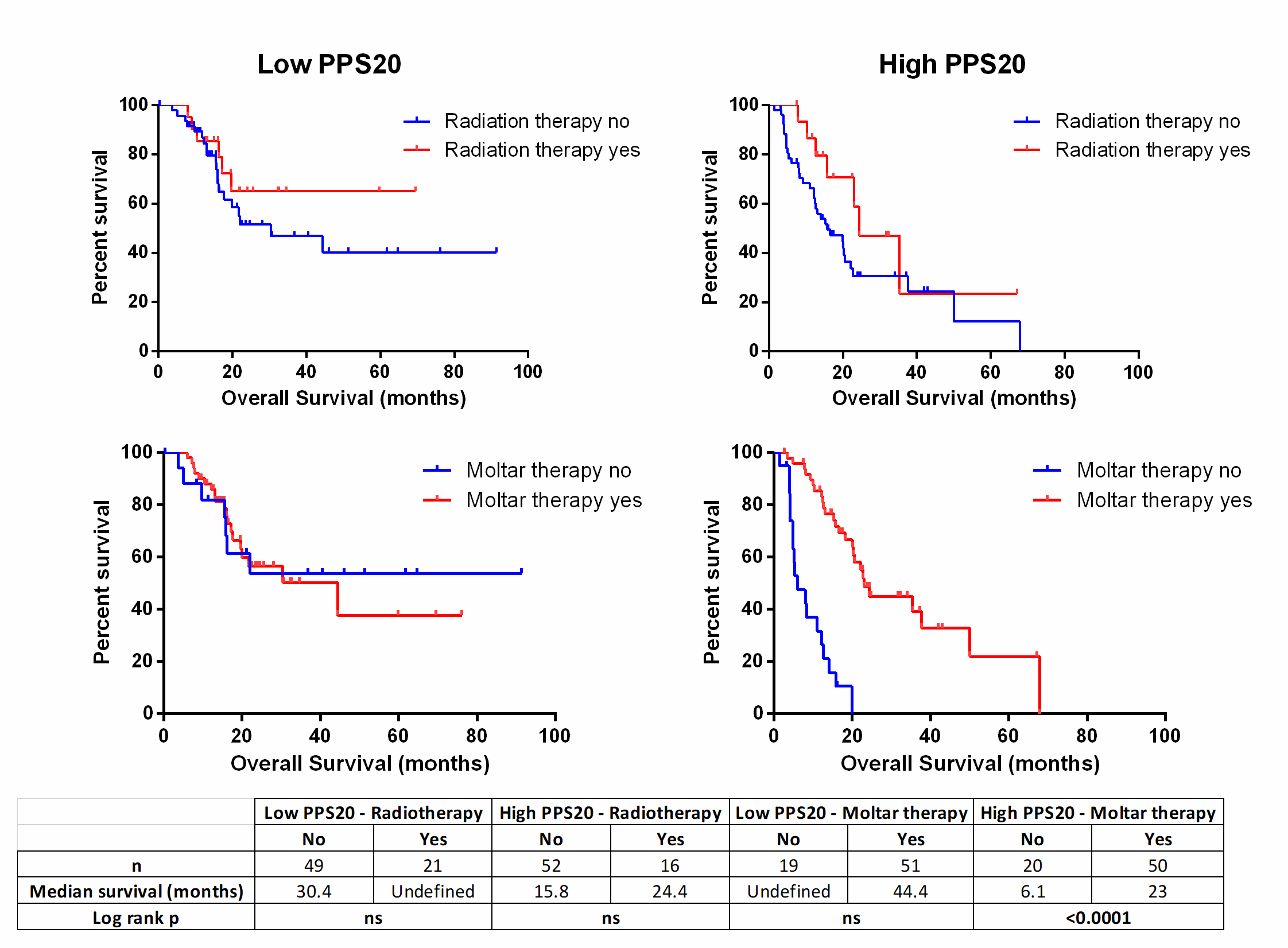

Supplement: S5 Fig — Kaplan Meier graphs stratified by PPS20 in TCGA PAAD comparing patients who received and did not receive radiation therapy and molecular targeted therapy. Statistics are shown below the figure. (TIF) [file pone.0231835.s005.tif]

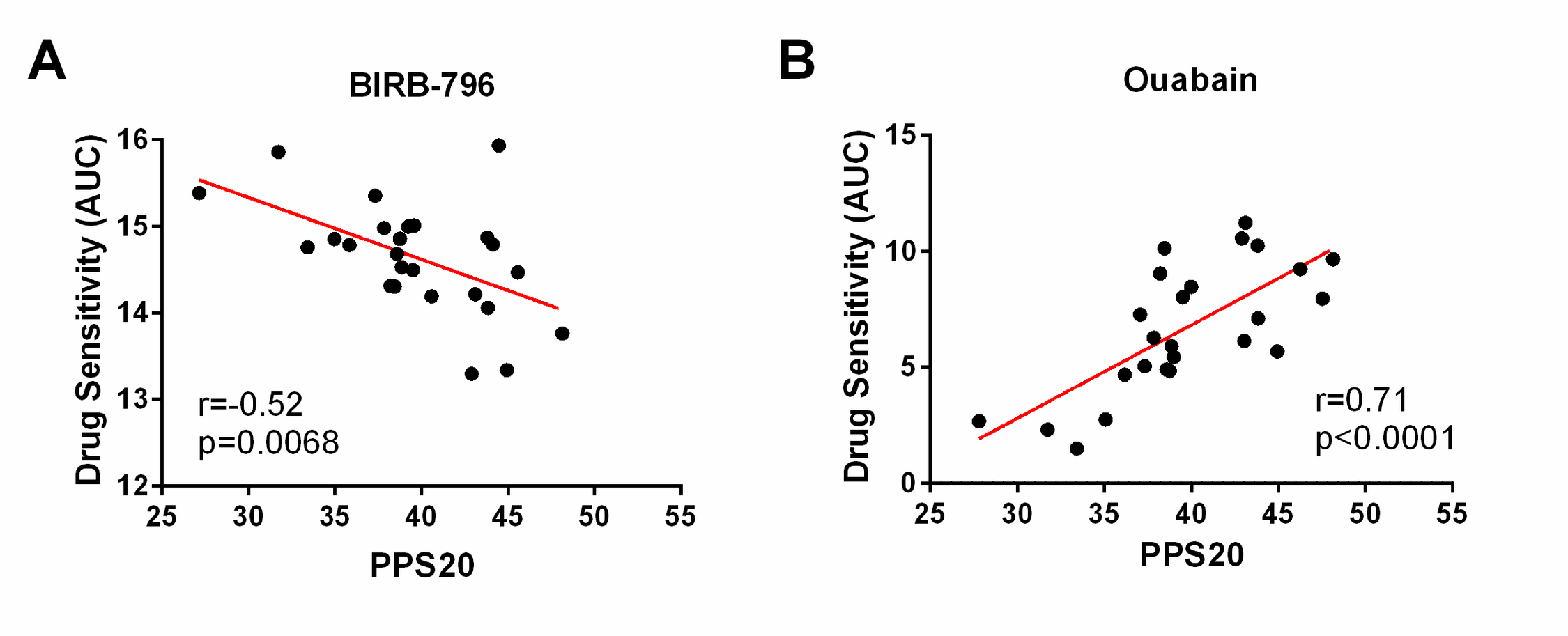

Supplement: S6 Fig — BIRB-796 sensitivity is negatively correlated with PPS20 and can preferentially target cells with high PPS20 (A). Ouabain shows the opposite pattern (B). (TIF) [file pone.0231835.s006.tif]

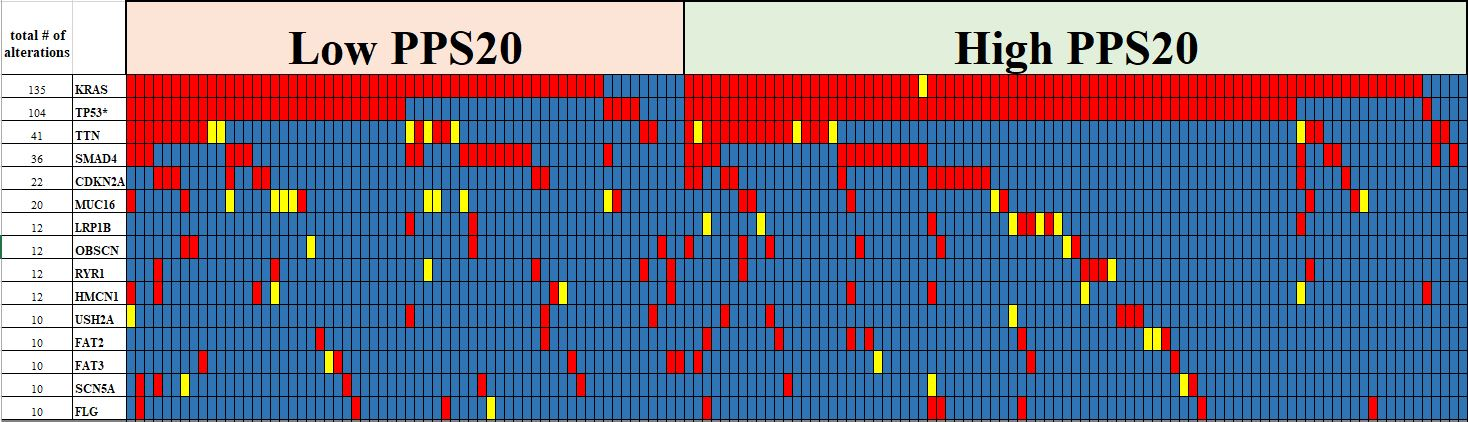

Supplement: S7 Fig — The genes which have been altered in at least 10 patients are shown. Chi-squared test with Yates' continuity correction was performed for each gene (synonymous variants excluded). Blue: No alterations, Yellow: Synonymous variants, Red: Nonsynonymous variants. * indicates a p value smaller than 0.05. (TIF) [file pone.0231835.s007.tif]
