# Supplementary material for: A novel 20-gene prognostic score in pancreatic adenocarcinoma
Source: PLoS One. 2020 Apr 20;15(4):e0231835. doi: 10.1371/journal.pone.0231835 (PMC7170253; doi:10.1371/journal.pone.0231835)
Supplement: S1 Table — (DOCX) [file pone.0231835.s008.docx]

**Table S1: Genes involved in PPS20**

|  |  | **GSE21501** | | **ICGC PACA CA** | | **ICGC PACA AU** | | **TCGA** | |
| --- | --- | --- | --- | --- | --- | --- | --- | --- | --- |
|  |  |  | |  | |  | |  | |
| **Gene Symbol** | **Official Full Name** | **HR*** | **P*** | **HR*** | **P*** | **HR*** | **P*** | **HR*** | **P*** |
| STX16 | syntaxin 16 | 1.77 | **0.0031** | 1.50 | **0.0207** | 1.89 | **0.0028** | 0.56 | **0.0077** |
| ERRFI1 | ERBB receptor feedback inhibitor 1 | 1.30 | **0.0113** | 1.31 | **0.0011** | 1.76 | **0.0005** | 1.11 | 0.2862 |
| TRIO | trio Rho guanine nucleotide exchange facto | 1.51 | **0.0184** | 1.73 | **0.0075** | 1.79 | **0.0150** | 1.16 | 0.3292 |
| SLC20A1 | solute carrier family 20 member 1 | 1.41 | **0.0317** | 1.28 | **0.0032** | 1.83 | **0.0050** | 1.37 | **0.0037** |
| RAB7A | RAB7A, member RAS oncogene family | 2.28 | **0.0019** | 1.89 | **0.0147** | 1.20 | 0.4067 | 3.75 | **0.0006** |
| GSK3B | glycogen synthase kinase 3 beta | 2.54 | **0.0049** | 2.00 | **0.0162** | 0.95 | 0.8417 | 3.02 | **0.0002** |
| TFG | TRK-fused gene | 2.24 | **0.0079** | 1.65 | **0.0301** | 1.21 | 0.4527 | 2.47 | **0.0001** |
| EPS8 | epidermal growth factor receptor pathway substrate 8 | 1.46 | **0.0275** | 1.43 | **0.0209** | 1.49 | 0.0885 | 2.12 | **<0.0001** |
| LDHA | lactate dehydrogenase A | 1.49 | **0.0297** | 1.61 | **0.0020** | 1.23 | 0.2264 | 2.09 | **<0.0001** |
| MAP4K4 | mitogen-activated protein kinase kinase kinase kinase 4 | 2.20 | **0.0003** | 1.28 | 0.1273 | 2.88 | **0.0011** | 2.13 | **0.0003** |
| ARNTL2 | aryl hydrocarbon receptor nuclear translocator like 2 | 1.47 | **0.0063** | 1.15 | 0.1405 | 1.53 | **0.0047** | 1.75 | **<0.0001** |
| CADPS2 | Ca2+-dependent activator protein for secretion 2 | 0.44 | **0.0008** | 0.71 | **0.0036** | 0.69 | **0.0011** | 1.33 | 0.0666 |
| MIA3 | MIA SH3 domain ER export factor 3 | 0.50 | **0.0011** | 0.72 | **0.0179** | 0.54 | **0.0088** | 0.87 | 0.4866 |
| NDUFB2 | NADH:ubiquinone oxidoreductase subunit B2 | 0.46 | **0.0078** | 0.60 | **0.0112** | 0.42 | **0.0004** | 0.58 | **0.0071** |
| POLR3H | RNA polymerase III subunit H | 0.53 | **0.0155** | 0.64 | **0.0081** | 0.50 | **0.0126** | 0.59 | **0.0122** |
| KANK1 | KN motif and ankyrin repeat domains 1 | 0.75 | **0.0444** | 0.66 | **0.0013** | 0.55 | **0.0264** | 0.73 | **0.0269** |
| C2orf42 | chromosome 2 open reading frame 42 | 0.42 | **0.0018** | 0.57 | **0.0129** | 1.15 | 0.4330 | 0.34 | **0.0001** |
| PITPNA | phosphatidylinositol transfer protein alpha | 0.43 | **0.0025** | 0.55 | **0.0204** | 0.34 | **0.0006** | 0.23 | **<0.0001** |
| ZNF557 | zinc finger protein 557 | 0.68 | **0.0300** | 0.72 | **0.0187** | 0.64 | 0.1211 | 0.43 | **0.0003** |
| CBX7 | chromobox 7 | 0.70 | **0.0136** | 0.88 | 0.1913 | 0.53 | **0.0030** | 0.49 | **<0.0001** |

* Cox proportional hazards regression performed with OS
