# Supplementary material for: A novel 20-gene prognostic score in pancreatic adenocarcinoma
Source: PLoS One. 2020 Apr 20;15(4):e0231835. doi: 10.1371/journal.pone.0231835 (PMC7170253; doi:10.1371/journal.pone.0231835)
Supplement: S2 Table — (DOCX) [file pone.0231835.s009.docx]

| **Table S2 : Cox regression analyses of PPS20 and clinicopathological parameters -OS** | | | | | |
| --- | --- | --- | --- | --- | --- |
| **TCGA (n=56)** |  | **Univariate** | | **Multivariate** | |
| **Patient Characteristics** | **Nr.** | **HR*** | **P*** | **HR*** | **P*** |
| **Age** |  |  |  |  |  |
| Age ≤ 60 (ref.) | 17 | 1.271 | 0.543 |  |  |
| Age > 60 | 39 |  |  |  |  |
| **Pathological Stage**** |  |  |  |  |  |
| Stage 1A (ref.) | 1 | 1.941 | **0.02** | 1.575 | 0.113 |
| Stage 1B | 10 |  |  |  |  |
| Stage 2A | 10 |  |  |  |  |
| Stage 2B | 33 |  |  |  |  |
| Stage 3 | 1 |  |  |  |  |
| Stage 4 | 0 |  |  |  |  |
| Unknown | 1 |  |  |  |  |
| **Gender** |  |  |  |  |  |
| Male (ref.) | 31 | 0.9 | 0.769 |  |  |
| Female | 25 |  |  |  |  |
| **Grade***** |  |  |  |  |  |
| Well differentiated (ref.) | 15 | 2.342 | **0.001** | 1.734 | **0.037** |
| Moderately differentiated | 27 |  |  |  |  |
| Poorly differentiated or undifferentiated | 13 |  |  |  |  |
| Unknown | 1 |  |  |  |  |
| **Prior Malignancy** |  |  |  |  |  |
| No (ref.) | 50 | 1.359 | 0.319 |  |  |
| Yes | 6 |  |  |  |  |
| **PPS20** |  |  |  |  |  |
| Low PPS20 (ref.) | 33 | 3.665 | **0.001** | 2.958 | **0.006** |
| High PPS20 | 23 |  |  |  |  |

Due to lack of information regarding specific therapy target, patients who received molecular targeted therapy were excluded. Patients with residual tumor status R1 were excluded

* Cox proportional hazards regression performed with OS

** Treated as a continuous variable 1:Stage1B, 2:Stage 2A, 3:Stage 2B

*** Treated as a continuous variable 1:Well differentiated, 2:Moderately differentiated, 3:Poorly differentiated or undifferentiated
