# Supplementary material for: A novel 20-gene prognostic score in pancreatic adenocarcinoma
Source: PLoS One. 2020 Apr 20;15(4):e0231835. doi: 10.1371/journal.pone.0231835 (PMC7170253; doi:10.1371/journal.pone.0231835)
Supplement: S3 Table — A: Univariate Analyses. B: Multivariate Analyses (Backward Wald). (DOCX) [file pone.0231835.s010.docx]

| **Table S3A: Univariate Analyses** | | |  |  |
| --- | --- | --- | --- | --- |
| **GSE62452 (n=65)** | **Nr.** | **HR*** | **P*** | **95% CI** |
| **PPS20** |  |  |  |  |
| High | 33 | **2.478** | **0.004** | **1.340-4.585** |
| Low | 32 |  |  |  |
| **Chen Signature** |  |  |  |  |
| High | 33 | **2.107** | **0.016** | **1.146-3.872** |
| Low | 32 |  |  |  |
| **Yan Signature** |  |  |  |  |
| High | 33 | 1.739 | 0.059 | 0.979-3.090 |
| Low | 32 |  |  |  |
| **Shi Signature** |  |  |  |  |
| High | 33 | 1.058 | 0.844 | 0.602-1.859 |
| Low | 32 |  |  |  |
| **Grade***** |  |  |  |  |
| G1 | 2 | **1.845** | **0.043** | **1.020-3.338** |
| G2 (ref.) | 32 |  |  |  |
| G3 | 29 |  |  |  |
| G4 | 1 |  |  |  |
| Gx | 1 |  |  |  |
| **Stage**** |  |  |  |  |
| Stage 1B | 4 | 1.386 | 0.066 | 0.979-1.963 |
| Stage 2A | 10 |  |  |  |
| Stage 2B | 35 |  |  |  |
| Stage 3 | 10 |  |  |  |
| Stage 4 | 3 |  |  |  |
| Stage 4A | 2 |  |  |  |
| Stage 4B | 1 |  |  |  |

*Cox proportional hazards regression performed with OS

**Stage was treated as continuous variable 1: Stage 2A, 2: Stage 2B, 3: Stage 3, 4: Stage 4, 4A &4B

*** G1 G4 & Gx were not included in the analyses

| **Table S3B: Multivariate Analyses (Backward Wald)** | | | | |
| --- | --- | --- | --- | --- |
| **GSE62452 (n=65)** | | **HR*** | **P*** | **95% CI** |
| **Step 1** | PPS20 | 1.432 | 0.132 | 0.898-2.283 |
|  | Chen Signature | 0.944 | 0.806 | 0.598-1.492 |
|  | Grade*** | 1.697 | 0.091 | 0.919-3.132 |
|  |  |  |  |  |
| **Step 2** | PPS20 | **1.371** | **0.048** | **1.003-1.874** |
|  | Grade*** | 1.685 | 0.093 | 0.916-3.099 |

*Cox proportional hazards regression performed with OS
