# Supplementary material for: A novel 20-gene prognostic score in pancreatic adenocarcinoma
Source: PLoS One. 2020 Apr 20;15(4):e0231835. doi: 10.1371/journal.pone.0231835 (PMC7170253; doi:10.1371/journal.pone.0231835)
Supplement: S4 Table — A: Univariate Analyses. B: Multivariate Analyses (Backward Wald). (DOCX) [file pone.0231835.s011.docx]

**Table S4A: Univariate Analyses**

| **GSE79668 (n=51)** | **Nr.** | **HR*** | **P*** | **95% CI** |
| --- | --- | --- | --- | --- |
| **PPS20** |  |  |  |  |
| High | 26 | **1.836** | **<0.0001** | **1.343-2.510** |
| Low | 25 |  |  |  |
| **Chen Signature** |  |  |  |  |
| High | 26 | **1.713** | **0.001** | **1.235-2.375** |
| Low | 25 |  |  |  |
| **Yan Signature** |  |  |  |  |
| High | 26 | **1.388** | **0.032** | **1.028-1.873** |
| Low | 25 |  |  |  |
| **Shi Signature** |  |  |  |  |
| High | 29 | **1.724** | **0.001** | **1.248-2.382** |
| Low | 22 |  |  |  |
| **AJCC Stage**** |  |  |  |  |
| Undetermined | 1 | 1.001 | 0.305 | 0.999-1.002 |
| IA | 2 |  |  |  |
| IB | 7 |  |  |  |
| IIA | 4 |  |  |  |
| IIB | 31 |  |  |  |
| III | 5 |  |  |  |
| IV | 1 |  |  |  |
| **Gender** |  |  |  |  |
| Male (ref.) | 32 | 0.899 | 0.501 | 0.659-1.226 |
| Female | 19 |  |  |  |
| **Diabetes Status** |  |  |  |  |
| No (ref) | 29 | 1.307 | 0.382 | 0.717-2.384 |
| Yes | 22 |  |  |  |
| **Age** |  |  |  |  |
| Age ≤ 60 (ref.) | 18 | 1.34 | 0.357 | 0.719-2.498 |
| Age > 60 | 33 |  |  |  |
| **T Stage**** |  |  |  |  |
| T1 | 1 | 0.752 | 0.156 |  |
| T2 | 12 |  |  |  |
| T3 | 31 |  |  |  |
| T4 | 2 |  |  |  |
| **N Stage** |  |  |  |  |
| N0 (ref.) | 14 | 0.833 | 0.297 |  |
| N1 | 37 |  |  |  |

*Cox proportional hazards regression performed with OS

**TNM Staging was converted to AJCC Stage via AJCC Cancer Staging Manual 8^th^ edition and utilized as continuous variables 1: IA&IB, 2: IIA&IIB, 3: III.

**Table S4B: Multivariate Analyses (Backward Wald)**

| **GSE79668 (n=51)** | | **HR*** | **P*** | **95% CI** |
| --- | --- | --- | --- | --- |
| **Step 1** | PPS20 | 1.585 | 0.049 | 1.002-2.508 |
|  | Chen Signature | 1.051 | 0.842 | 0.647-1.706 |
|  | Yan Signature | 1.028 | 0.874 | 0.728-1.453 |
|  | Shi Signature | 1.481 | 0.043 | 1.012-2.168 |
|  |  |  |  |  |
| **Step 2** | PPS20 | 1.609 | 0.027 | 1.055-2.454 |
|  | Chen Signature | 1.045 | 0.858 | 0.647-1.687 |
|  | Shi Signature | 1.491 | 0.034 | 1.030-2.159 |
|  |  |  |  |  |
| **Step 3** | PPS20 | 1.648 | 0.003 | 1.186-2.290 |
|  | Shi Signature | 1.511 | 0.018 | 1.074-2.126 |

*Cox proportional hazards regression performed with OS
