# Supplementary material for: A novel 20-gene prognostic score in pancreatic adenocarcinoma
Source: PLoS One. 2020 Apr 20;15(4):e0231835. doi: 10.1371/journal.pone.0231835 (PMC7170253; doi:10.1371/journal.pone.0231835)
Supplement: S5 Table — (DOCX) [file pone.0231835.s012.docx]

**Table S5: Gene sets enriched in high PPS20 tumors**

|  | **NES*** | | | |
| --- | --- | --- | --- | --- |
|  | **TCGA** | **PACA-CA** | **PACA-AU** | **GSE71729** |
| GO_KERATINOCYTE_DIFFERENTIATION | 1.37 | 1.52 | 2.13 | 2.34 |
| GO_SKIN_DEVELOPMENT | 1.4 | 1.54 | 2.02 | 2.26 |
| GO_EPIDERMIS_DEVELOPMENT | 1.58 | 1.65 | 1.96 | 2.38 |
| GO_FORMATION_OF_PRIMARY_GERM_LAYER | 1.54 | 1.71 | 1.93 | 1.97 |
| GO_ENDODERM_DEVELOPMENT | 1.62 | 1.89 | 1.83 | 1.73 |

* NES: Normalized enrichment score
