# Supplementary material for: A novel 20-gene prognostic score in pancreatic adenocarcinoma
Source: PLoS One. 2020 Apr 20;15(4):e0231835. doi: 10.1371/journal.pone.0231835 (PMC7170253; doi:10.1371/journal.pone.0231835)
Supplement: S6 Table — (DOCX) [file pone.0231835.s013.docx]

**Table S6: Gene sets enriched in low PPS20 tumors**

|  | **NES*** | | | |
| --- | --- | --- | --- | --- |
|  | **TCGA** | **PACA-CA** | **PACA-AU** | **GSE71729** |
| GO_DIGESTION | -1.56 | -2 | -2.61 | -2.05 |
| GO_PROTEIN_ACTIVATION_CASCADE | -1.54 | -1.72 | -1.87 | -2.29 |
| GO_POTASSIUM_CHANNEL_ACTIVITY | -1.51 | -1.6 | -1.48 | -1.53 |
| GO_POTASSIUM_ION_TRANSPORT | -1.51 | -1.69 | -1.39 | -1.63 |

* NES: Normalized enrichment score
