# Supplementary material for: A novel 20-gene prognostic score in pancreatic adenocarcinoma
Source: PLoS One. 2020 Apr 20;15(4):e0231835. doi: 10.1371/journal.pone.0231835 (PMC7170253; doi:10.1371/journal.pone.0231835)
Supplement: S7 Table — A: 5 drugs targeting cells with ‘high PPS20’. Negative correlation between PPS20 and drug cytotoxicity data (AUC) shows drugs targeting ‘high PPS20’ group. B: 5 drugs targeting cells with ‘low PPS20’. Positive correlation between PPS20 and drug cytotoxicity data (AUC) shows drugs targeting ‘low PPS20’ group. (DOCX) [file pone.0231835.s014.docx]

**Table S7A:** 5 drugs targeting cells with ‘high PPS20’. Negative correlation between PPS20 and drug cytotoxicity data (AUC) shows drugs targeting ‘high PPS20’ group.

| **Drugs** | **Pearson** | **p value** | **Target / Activity of compound** | |
| --- | --- | --- | --- | --- |
| BIRB-796 | -0.504 | 0.01024 | inhibitor of p38 MAPK |  |
| tivozanib | -0.453 | 0.01548 | inhibitor of VEGFRs |  |
| L-685458 | -0.417 | 0.04286 | inhibitor of gamma-secretase |  |
| BRD-A05715709 | -0.544 | 0.04431 | putative inhibitor of IDH1 R132H |  |
| BRD-K48477130 | -0.478 | 0.04488 | screening hit |  |

**Table S7B:** 5 drugs targeting cells with ‘low PPS20’. Positive correlation between PPS20 and drug cytotoxicity data (AUC) shows drugs targeting ‘low PPS20’ group.

| **Drugs** | **Pearson** | **p value** | **Target / Activity of compound** |
| --- | --- | --- | --- |
| ouabain | 0.722 | 0.00003 | cardiac glycoside; inhibitor of the Na+/K+-ATPase |
| SCH-79797 | 0.621 | 0.00006 | antagonist of proteinase-activated receptor 1 (PAR1) |
| teniposide | 0.71 | 0.00097 | inhibitor of topoisomerase II |
| pevonedistat | 0.602 | 0.00145 | inhibitor of Nedd-8 activating enzyme |
| clofarabine | 0.514 | 0.00263 | inducer of DNA damage |
